# Tracking *Arachis hypogaea* Allergen in Pre-Packaged Foodstuff: A Nanodiamond-Based Electrochemical Biosensing Approach

**DOI:** 10.3390/bios12060429

**Published:** 2022-06-18

**Authors:** Maria Freitas, André Carvalho, Henri P. A. Nouws, Cristina Delerue-Matos

**Affiliations:** REQUIMTE/LAQV, Instituto Superior de Engenharia do Porto, Instituto Politécnico do Porto, Rua Dr. António Bernardino de Almeida 431, 4200-072 Porto, Portugal; a_carvalho_16@hotmail.com (A.C.); cmm@isep.ipp.pt (C.D.-M.)

**Keywords:** food allergen, Ara h 1, nanodiamonds, scanning electron microscopy, energy dispersive spectroscopy, voltammetric immunosensor, screen-printed electrode, peanut allergy, foodstuff

## Abstract

The present work reports a nanodiamond-based voltammetric immunosensing platform for the analysis of a food allergen (Ara h 1) present in peanuts (*Arachis hypogaea*). The possibility of the usage of nanodiamonds (*d* = 11.2 ± 0.9 nm) on screen-printed carbon electrodes (SPCE/ND) in a single-use two-monoclonal antibody sandwich assay was studied. An enhanced electroactive area (~18%) was obtained and the biomolecule binding ability was improved when the 3D carbon-based nanomaterial was used. The antibody-antigen interaction was recognized through the combination of alkaline phosphatase with 3-indoxyl phosphate and silver ions. Linear Sweep Voltammetry (LSV) was applied for fast signal acquisition and scanning electron microscopy (SEM) and energy dispersive spectroscopy (EDS) support the voltammetric approach and confirm the presence of silver particles on the electrode surface. The proposed immunosensor provided a low limit of detection (0.78 ng·mL^−1^) and highly precise (RSD < 7.5%) and accurate results. Quantification of Ara h 1 in commercial foodstuffs (e.g., crackers, cookies, protein bars) that refer to the presence of peanuts (even traces) on the product label was successfully achieved. The obtained data were in accordance with recovery results (peanut addition, %) and the foodstuff label. Products with the preventive indication “may contain traces” revealed the presence of peanuts lower than 0.1% (*m*/*m*). The method’s results were validated by comparison with an enzyme-linked immunosorbent assay. This allows confident information about the presence of allergens (even at trace levels) that leads to profitable conditions for both industry and consumers.

## 1. Introduction

Food labeling and allergen content for pre-packaged commercial products are required by legislation to ensure manufacturers’ compliance and consumer protection while enabling citizens to access comprehensive information [1,2]. A priority list of major allergens, substances, and/or products thereof is regulated by EU law [1] that is in line with the FDA [2], which sets out mandatory specifications and precautionary advertisements. Despite the efforts of regulatory agencies, the quantification of ingredients is still largely disregarded. Furthermore, hidden substances or allergen cross-contamination are a pressing concern for quality management in food chains with the assessment of (extremely) low quantities as a huge challenge [3]. Hence, foodstuff labels must declare trace amounts to prevent hypersensitive individuals and/or consumers from undesirable symptoms and reactions [4]. Allergen avoidance is the best option for food-allergic consumers because of mislabeled or unclear product labels. Rescue medication (e.g., antihistamines) is an effective treatment in case of severe reactions (e.g., anaphylaxis) from accidental exposure or unawareness of food ingredients [5].

Epidemiological data report that a significant burden on peanut-allergic patients has a rising prevalence, especially in children, representing a worrying condition for public health [6]. Peanuts (*Arachis hypogaea*) are a source of highly allergic proteins that, according to the WHO/IUIS Allergen Nomenclature Sub-Committee, harbor 18 allergens and distinct isoforms [7], with Ara h 1, Ara h 2, and Ara h 3 being considered the major and most prevalent of these allergens. In addition, Ara h 6 has been referred to as a potential allergen to be studied for the detection of traces of peanut in foodstuff due to its similarity to Ara h 2 [8]. In the Mediterranean region, Ara h 9 has been described as having a high prevalence [9]. Nonetheless, as Ara h 1 is recognized by serum IgE in over 90% of peanut-allergic patients (hence considered a major allergen), is a thermostable protein and resistant to digestion in the human gastrointestinal tract, it is a specific analyte that is suitable for identifying the presence of peanuts in food products [10].

Portable screening platforms for in situ analysis of allergens (e.g., (bio)sensors) can assure accurate foodstuff labeling, being effective alternatives to classical standard methods (ELISA, PCR, RT-PCR) [11]. Disposable biosensors have proven their practical usefulness by providing rapid results and simplified analysis methodologies for assessing food safety and quality [12]. Regarding affordable biosensing devices for food allergen concerns, immunosensors are prominently featured [13,14]. Hence, tracking peanuts in pre-packaged foods using electrochemical immunoassays by employing self-assembled monolayers and/or nano- and micromaterials has been accomplished and described for Ara h 1 [15,16,17,18,19]. The reported strategies contribute to the progress of miniaturized and portable analytical platforms and include an impedance immunosensor developed using conventional gold electrodes functionalized with a self-assembled monolayer (11-mercaptoundecanoic acid) [15], a functionalized single-walled carbon nanotubes (SWCNT)-based junction sensor (gold assembly on silicon wafers) [16], an amperometric magnetoimmunosensing platform using carboxylic-acid-modified Magnetic Beads (MBs) using bare screen-printed carbon electrodes (SPCE) [17], and a gold-nanoparticle-coated SPCE sandwich-type immunosensor, able to detect Ara h 1 in samples containing 0.1% (*m*/*m*) of peanut, applied to the analysis of cookies and chocolate [18]. Recently, quantum dots were employed as the electroactive label to develop a voltammetric immunosensor that employed bare SPCEs as a simple and non-modified transducer. In this work, through immunological interaction, the target analyte was detected in commercial organic farming cookies (down to 0.05% (*m*/*m*) of peanut) [19].

Biosensing platforms resort to technological breakthroughs to improve figures of merit, with interdisciplinary approaches deserving particular attention. The overarching goals in this field are the integration of nanoscale materials to enhance sensitivity [20] and the use of selective recognition elements to maximize the analytical performance of the analysis of complex matrices. Regarding the nanoscale materials, diamond (especially in its nanostructured form—nanodiamond) is a promising carbon-based material with potential applications in electrochemical biosensing strategies and bioapplications [21,22,23]. Moreover, due to the 3D structure, unique characteristics such as high biocompatibility, easy bioconjugation, and chemical and optical properties (e.g., inertness, stability, wide potential window, transparency), NDs have attracted considerable attention in biosensor/biochip applications for point-of-care testing [24]. Accordingly, nanodiamond-based electrochemical (immuno)assays are a noteworthy approach for bioanalytical purposes, antibody immobilization, and biosensor applications [25,26,27,28].

Considering the threshold values for peanut-allergic individuals in food products attained by No Observed Adverse Effect Level (NOAEL) determinations (ranging from 0.25 to 10 mg·kg^−1^ of whole peanut) [29] and the need for quality assurance, the present work describes an electrochemical nanodiamond-based immunosensor based on a screen-printed carbon electrode (SPCE/ND) for accurate analysis of low quantities and relevant doses of the peanut allergen Ara h 1 in pre-packaged commercial products. For electroanalytical purposes, the electrical conductivity and the stable dispersion of nanodiamonds in an aqueous solution are valued characteristics for efficient platform nanostructuration [30]. Here, a suitable biomodification of the single-use SPCE/ND with anti-Ara h 1 capture antibodies was reached by physisorption. The target analyte was analyzed in a sandwich-type biosensing approach where a streptavidin-labeled enzyme bound to a specific biotinylated detection antibody through affinity was used. The biochemical interaction was electrochemically detected after the formation of metallic silver and the voltammetric signal was acquired by applying an anodic potential scan (Linear Sweep Voltammetry (LSV)). The optimized sensing platform was characterized by Scanning Electron Microscopy (SEM) and Energy Dispersive Spectroscopy (EDS). Elemental analysis confirmed the presence of silver particles on the sensor’s surface. The accurate and highly sensitive determination of Ara h 1 was verified along with the applicability of the developed sensing strategy to commercial products using a rapid and user-friendly process (hands-on time ≤ 10 min). The accuracy of the results was assessed using a conventional ELISA kit.

## 2. Materials and Methods

### 2.1. Instrumentation and Reagents

FEI Quanta 400FEG ESEM/EDAX Genesis X4 M equipment (Hillsboro, OR) was used to obtain the Scanning Electron Microscopy (SEM) images and the energy dispersive spectroscopy (EDS), with a SUTW SAPHIRE detector, was operated at 15 kV with a system resolution of 132.19. These analyses were performed at the “Centro de Materiais da Universidade do Porto (CEMUP)”. Electrochemical measurements were performed using Screen-Printed Carbon Electrodes (SPCE, carbon working electrode (WE, d = 4 mm), carbon counter electrode (CE), and silver pseudo-reference electrode (RE), DRP-110, Metrohm DropSens), interfaced through a specific connector (DRP-CAC, Metrohm DropSens, Oviedo, Spain) to an Autolab PGSTAT101 potentiostat—galvanostat controlled by the NOVA software (v.1.10, Metrohm Autolab, Utrecht, The Netherlands). A chopper (Moulinex) and a centrifuge (Megafuge 16R Thermo-Heraeus, Thermo Fisher Scientific, Osterode am Harz, Germany) were used for sample preparation. Ara h 1 ELISA kit (EPC-AH1-1), capture antibody (CAb, monoclonal anti-Ara h 1, 2C12), purified natural Ara h 1 (Ara h 1 standard, ST-AH1), and detection antibody (DAb, monoclonal anti-Ara h 1, Biotin 2F7) were obtained from Indoor Biotechnologies. ELISA was carried out using a multi-mode microplate reader (Synergy HT W/TRF, BioTek Instruments, Winooski, VT, USA) with a Gen5 Version (2.0 data analysis software, BioTek Instruments, Winooski, VT, USA). Albumin from bovine serum (BSA), 3-indoxyl phosphate (3-IP), nanodiamonds (NDs, nanopowder), nitric acid (HNO_3_), potassium chloride (KCl), silver nitrate, and tris(hydroxymethyl)aminomethane (Tris) were purchased from Sigma-Aldrich. Streptavidin-alkaline phosphatase (S-AP) was acquired from Thermo Fisher Scientific.

Solutions of BSA and CAb were prepared in T1 (Tris-HNO_3_, 0.1 M, pH 7.2, Tris buffer); Ara h 1, DAb, S-AP solutions were prepared in T2 (Tris buffer, containing 1.0% BSA (*m*/*v*)). The solution containing 3-IP (1.0 × 10^−3^ M) and silver nitrate (4.0 × 10^−4^ M) was prepared in T3 (Tris 0.1 M (pH 9.8 + Mg(NO_3_)_2_ 2.0 × 10^−2^ M)). Ara h 1 extraction from commercial food samples was carried out using an extraction buffer (Tris buffer, pH 8.5).

### 2.2. Sample Preparation

Ingredients and food products were bought in local supermarkets to evaluate the immunosensor’s performance: 1—wheat flour, 2—oat, 3—lupine, 4—pea, 5—soybean, 6—almond, 7—hazelnut, 8—energy bar (no peanut), 9—biscuit (no peanut), 10—water cracker, 11—oatmeal cookie, 12—whole-grain cereal, 13—granola, 14—muesli, 15—protein bar (5% peanut), 16—protein bar (12% peanut), 17—peanut and pineapple cookie (8% peanut). The extraction procedure was performed as recommended by the Ara h 1 standard supplier (Indoor Biotechnologies). Briefly, 1 g of the sample was mixed with 10 mL of the extraction buffer, vortexed for 5 s, incubated for 15 min at 60 °C, centrifuged at 2500 rpm for 20 min, and stored at −20 °C until use. Samples were diluted in T2 as follows: 100× for samples without peanut allergen or containing traces and 1000× for samples with known peanut quantities.

### 2.3. Immunosensor Construction and Electrochemical Detection

**Figure 1:**
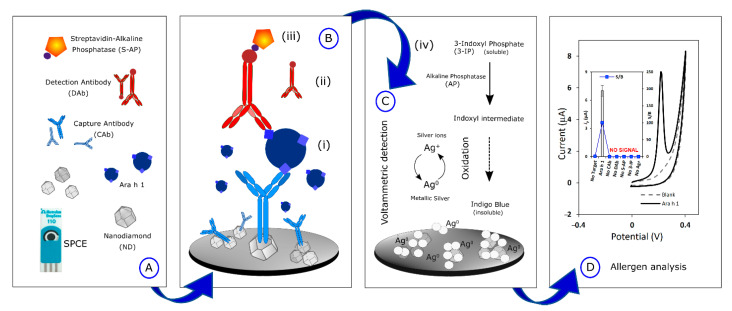
Schematic illustration of the analysis of *Arachis hypogaea* allergen. (**A**) SPCE nanostructuration with NDs, (**B**) Sandwich-type voltammetric immunosensor construction, (**C**) Metalloenzymatic reaction, (**D**) Linear Sweep voltammograms of the silver particles (inset: evaluation of the immunoassay performance in the presence of the target analyte (Ara h 1) and the absence of several of the assay´s reagents.

A schematic representation of the immunosensor’s construction and the analytical signal acquisition is presented in Figure 1.

(A) The SPCEs were nanostructured by drop-casting a 15-µL aliquot of NDs (100 µg·mL^−1^, previously dispersed in H_2_O and ultrasonicated for 1h to improve the nucleation density). Then, 10 µL of CAb (10 µg·mL^−1^) was placed on the SPCE and incubated overnight, at 2–8 °C, in a humidity chamber (immobilization through physisorption).
(B) The sandwich-type electrochemical immunoassay consisted of sequential incubation steps: (i) Ara h 1 standard allergen/food sample extract (40 µL, 30 min), (ii) DAb (40 µL, 250×, 60 min), (iii) S-AP (40 µL, 20,000×, 30 min).
(C) The enzymatic reaction took place by adding (iv) a 40 µL-aliquot of a mixture containing (iv) 3-IP (1.0 × 10^−3^ M) and AgNO_3_ (4.0 × 10^−4^ M), that reacted for 20 min. Silver ions were firstly reduced to metallic silver and subsequently, the silver particles were co-deposited with an insoluble component—indigo blue. Washing steps were performed using T1 (before steps i, ii, and iii) and T3 (before step iv).
(D) The electrochemical (oxidation) analysis of the deposited silver was carried out by LSV (potential range from −0.03 V to +0.4 V, scan rate: 50 mV·s^−1^).

## 3. Results and Discussion

### 3.1. Electrode Surface Nanostructuration and Characterization

Tracking trace amounts of food allergens in foodstuff requires analytical tools, such as biosensors, whose detection limits allow the fulfillment of the intended objective. The versatility, size, portability, and small sample volume required to perform analyses using screen-printed electrodes (SPE) have demonstrated the success of the application of these transducers. Furthermore, the usage of carbon-based nanomaterials for SPE nanostructuration can improve the detection of the target analyte. Hence, bare SPCE were used in the present work to analyze Ara h 1 and the results were compared with nanostructured SPCEs to evaluate the signal enhancement. A sandwich-type assay was employed, based on specific antibodies that matched the allergen under study, and the signal-to-blank (S/B, Signal (S) for 250 ng·mL^−1^ Ara h 1 and Blank (B) in the absence of Ara h 1) ratio was used to select the optimum transducer surface. The electrochemical signal was recorded through the anodic stripping of the enzymatically generated metallic silver, after the sandwich-type immunoassay construction (as can be observed in Figure 1). In brief, the enzyme (alkaline phosphatase) hydrolyses the enzymatic substrate 3-IP into an indoxyl intermediate that is subsequently oxidized forming indigo blue. During this process, silver ions, from silver nitrate, are reduced into metallic silver and the Ag^0^ formed on the working electrode is directly proportional to the amount of Ara h 1. The formed silver nanoparticles (co-deposited with indigo blue) were re-oxidized by applying a voltammetric potential scan [31].

**Figure 2:**
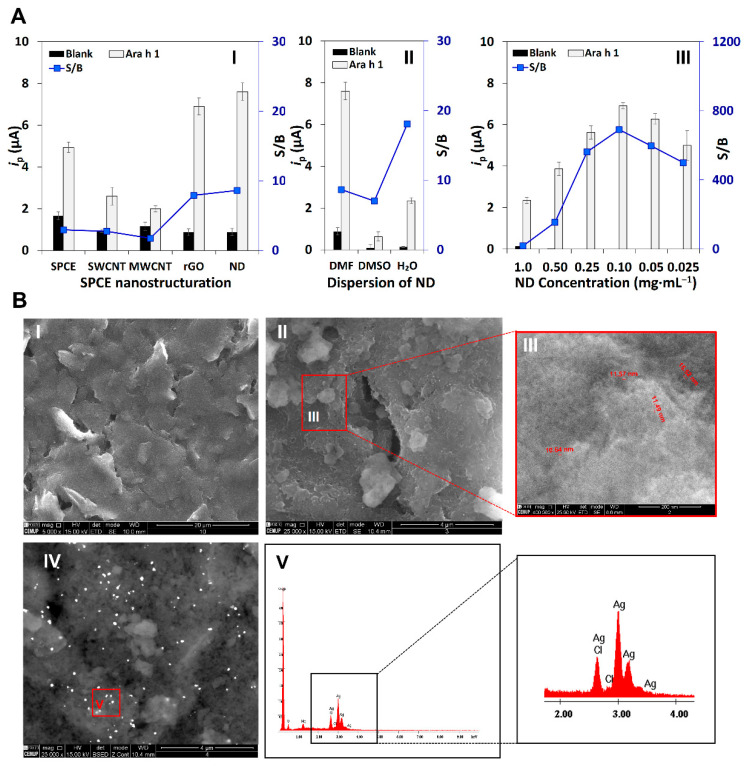
(**A**) **Voltammetric analysis:** (**I**) SPCE modified with carbon-based nanomaterials (SPCE, SPCE with single-walled nanotubes (SWCNT), multi-walled nanotubes (MWCNT), reduced graphene oxide (rGO), and nanodiamonds (ND)); (**II**) ND dispersed in distinct solvents (DMF, DMSO, and H_2_O); (**III**) ND concentration optimization. (**B**) **SEM and EDS analysis:** SEM images of (**I**) SPCE (scale bar: 20 µm); (**II**) SPCE/ND (scale bar: 4 µm), (**III**) NDs (scale bar: 200 nm), (**IV**) optimized assay (3IP-Ag^+^/S-AP/DAb/Ara h 1/CAb/ND modified SPCE; scale bar: 20 µm), and (**V**) EDS analysis of the optimized assay. Experimental conditions: Ara h 1 (0 and 250 ng·mL^−1^), 3-IP/Ag^+^ (1.0 × 10^−3^ M/4.0 × 10^−4^ M). Error bars are the standard deviation of three replicates.

Carbon-based nanomaterials with distinct dimensions (1D—single-walled nanotubes (SWCNT) and multi-walled nanotubes (MWCNT), 2D—reduced graphene oxide (rGO), 3D—nanodiamonds (ND)) were dispersed in DMF (1.0 mg·mL^−1^), drop-casted on the WE and their performance was evaluated. The obtained peak current intensities (*i*_p_) and S/B values are shown in Figure 2AI. As can be observed, the SPCE’s nanostructuration with ND revealed the highest S/B ratio and was thus selected to proceed with the studies. Distinct solvents that are typically employed for the dispersion of carbon-based nanomaterials (DMF, DMSO, and H_2_O) were tested, and the dispersion in H_2_O, which is much more environmentally friendly, provided the optimum result (Figure 2AII). Then, several ND concentrations were tested: 1.0, 0.50, 0.25, 0.10, 0.05, and 0.025 mg·mL^−1^, and based on the S/B ratio the best performance was reached for 0.10 mg·mL^−1^ (Figure 2AIII).

Distinct methods were employed for the WE surface characterization, including (i) electrochemical analysis—Cyclic Voltammetry (CV) and LSV for voltammetric evaluation and (ii) microscopy analysis—SEM and EDS for the nanostructured platform evaluation with elemental composition identification. The acquired SEM images of the SPCE and the SPCE/ND are displayed in Figure 2BI–III.

The nano-scale material (NDs) deposited on the transducer surface has a representative round shape (either spherical or elliptical), rough surfaces, and is broadly distributed on the SPCE’s WE, as can be observed in the electron microscopy image (Figure 2BIII). The average size of the material (*d* = 11.2 ± 0.9 nm) is in accordance with the supplier’s information (< 10 nm particle size, through TEM analysis). Hence, the easy platform structuration performed with NDs for the analysis of *Arachis hypogaea*, the efficient surface distribution, and the characteristic shape (3D) are outstanding properties for antibody immobilization/bioconjugation for the detection of the food allergen.

To estimate the electroactive surface area of the WE, a 5.0-µM [Fe(CN)_6_]^3−/4−^ solution was used in 0.1 mol·L^−1^ KCl and the effect of scan rate was studied by CV for both the bare (SPCE) and the nanostructured (SPCE/ND) electrode at scan rates varying from 0.01 to 0.1 V·s^−1^, obtaining clear cyclic voltammetric profiles. Then, the Randles–Ševčík equation was applied (*i*_p_ = (2.69 × 10^5^)AD^1/2^*n*^3/2^ν^1/2^C, where A is the electroactive surface area, D is the diffusion coefficient for [Fe(CN)_6_]^3−/4−^ (7.6 × 10^−6^ cm^2^·s^−1^), n is the number of electrons transferred (*n* = 1), ν is the scan rate, and C is the concentration of [Fe(CN)_6_]^3−/4−^ (5.0 mM). The plots between *i*_pa_ and ν^1/2^ and the recorded cyclic voltammograms are presented in Figure S1, and the calculated areas were 0.075 cm^2^ for the SPCE and 0.092 cm^2^ for the SPCE/ND. The 17.7-% increase for the SPCE/ND allows an enhanced electroactive area that increases the sensitivity of the analysis, thus suggesting that the probe can permeate into the packed nanostructured surface enhancing the electron charge transfer. This is in accordance with previously published works that reported the dispersion of NDs in acid conditions and the enhancement of the electroactive area [26,27]. However, in our approach, a more sustainable transducer nanostructuration was achieved by using an environmentally friendly dispersion of ND in H_2_O.

### 3.2. Evaluation of the Immunoassay’s Performance

Once the immunosensing platform was established, the evaluation of non-specific adsorptions and interactions was assessed using control assays (complete assay in the absence and presence of Ara h 1 (0 and 250 ng·mL^−1^)). In addition, the sensor’s evaluation of non-specific interactions was tested in the absence of the immunoreagents (CAb, DAb, S-AP) and the metalloenzymatic label (3-IP + AgNO_3_). The obtained data are shown in Figure S2A. Representative CV and LSV voltammograms, depicted in Figure S2B,C, show that in the absence of Ara h 1 no voltammetric peak related to silver re-oxidation was acquired. LSV was chosen among the voltammetric techniques because of its simplicity. These studies confirmed the correct performance of all reagents without interferences or undesirable adsorptions/interactions and the obtained data corroborate that the nanostructured SPCE/ND effectively provided an alternative option for the analysis of trace amounts of Ara h 1 as required by legislation. To fully characterize and confirm the final structure comprising 3IP-Ag+/S-AP/DAb/Ara h 1/CAb/ND modified SPCE, the SEM image related to the electrode surface was acquired (Figure 2BIV). The results support the voltammetric analysis, efficiently accomplished by the enzymatic generation of silver, as can be observed by the presence of dense particles on the electrode surface. In accordance, the EDS analysis (Figure 2BV) confirms that the dense bright deposits correspond to silver particles.

### 3.3. Optimization of Experimental Variables

The following experimental parameters of the sandwich immunoassay were optimized: (A) CAb concentration, (B) DAb dilution, (C) S-AP dilution, (D) Assay format, and (E) Assay time. A detailed description of these studies is included in the Supplementary Material of this article and the obtained data are shown in Figure S3. The tested and selected parameters are presented in Table 1. In short, the best results were obtained using the following experimental conditions: CAb 10 µg·mL^−1^, DAb 250× dilution, S-AP 200,000× dilution, 3-IP 1.0 × 10^−3^ M, silver nitrate 4.0 × 10^−4^ M, in a step-by-step sandwich-type immunoassay, with a total assay time of 2 h 20 min.

### 3.4. Analytical Performance, Storage Stability, and Selectivity of the Immunosensor

**Figure 3:**
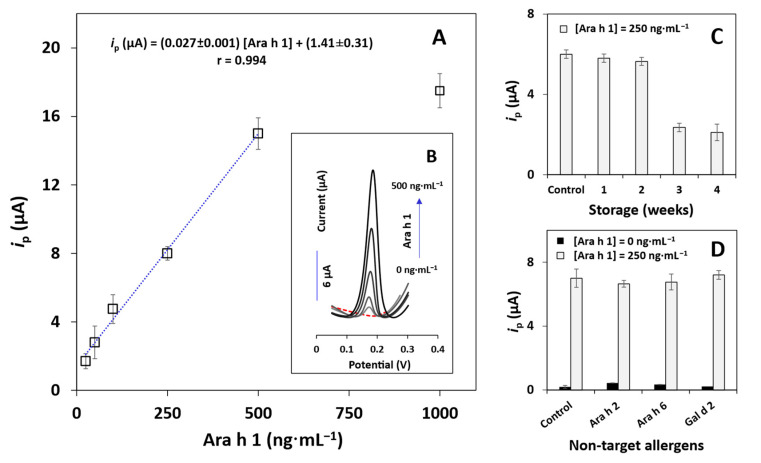
(**A**) Calibration plot for the analysis of Ara h 1 (*i*_p_ vs. Ara h 1 concentration) using the SPCE/ND immunosensor. (**B**) Representative linear sweep voltammograms of [Ara h 1]: 0, 25, 50, 100, 250 and 500 ng·mL^−1^). (**C**) Storage stability of the sensor (Ara h 1: 250 ng·mL^−1^) after 1 day (control) and for 4 weeks. (**D**) Selectivity and interference studies (Ara h 1 (0 and 250 ng·mL^−1^), non-target allergens: Ara h 2 (250 ng·mL^−1^), Ara h 6 (5.0 ng·mL^−1^), Gal d 2 (180 mg·mL^−1^)). Other conditions: CAb: 10 µg·mL^−1^, DAb: 250×, S-AP: 200,000×, 3-IP/Ag^+^: 1.0 × 10^−3^ M/4.0 × 10^−4^ M. LSV parameters: potential range from −0.03 V to +0.4 V, scan rate: 50 mV·s^−1^. Error bars are the standard deviations of three replicates.

The analytical performance of the SPCE/ND immunosensor was evaluated using the previously optimized parameters. The linear relationship between the i_p_ of the silver re-oxidation and Ara h 1 concentration was evaluated between 10 and 1000 ng·mL^−1^. For concentrations below 25 ng·mL^−1^ the obtained results were similar to the blank signal while for concentrations above 500 ng·mL^−1^ linearity was no longer observed (i.e., the signal leveled off). Thus, the linear range between i_p_ and Ara h 1 concentration was established between 25 and 500 ng·mL^−1^ (Figure 3A). The following regression equation was obtained: i_p_ (µA) = (0.027 ± 0.001) [Ara h 1] + (1.41 ± 0.31) (r = 0.994, n = 5, sensitivity = 0.29 μA·mL·ng^−1^·cm^−2^). Examples of the obtained voltammograms within the linear range are present in Figure 3B. The limits of detection (LOD = 3 × s/m) and quantification (LOQ = 10 × s/m) were estimated from the calibration plot (s is the standard deviation of the blank and m is the slope). A LOD of 0.78 ng·mL^−1^ and a LOQ of 2.6 ng·mL^−1^ were obtained, demonstrating that the established method can efficiently be employed to track and quantify the presence of peanuts in commercial products (the threshold level established for peanut allergen is up to 0.25 mg of protein [29]) and thus prevent inconvenient symptoms and reactions. The coefficient of variation of the method (V_x0_ = 9.6%) demonstrated adequate precision.

The precision of the immunosensor’s response was evaluated using a 250-ng·mL^−1^ Ara h 1 solution. The repeatability and reproducibility were assessed by three successive inter-electronic measurements, in triplicate, on different days. Relative standard deviations (RSD) of 7.3% and 4.9% were obtained, respectively, indicating precise results.

Furthermore, since storage stability affects the product’s usability in commercial applications, several SPCE/ND platforms were biomodified with CAb, stored in a moist environment at 2–8 °C, and its response toward a 250-ng·mL^−1^ Ara h 1 solution was evaluated on the day after preparation (control) and during four consecutive weeks. The obtained i_p_ values (Figure 3C) indicated a stability of 2 weeks, with no significant signal loss. However, after 3 weeks a significant decrease in the i_p_ was observed. Consequently, the SPCE/ND/CAb platform can be used within a 2-week period after preparation and adequate storage.

The selectivity and possible interferences of the assay were evaluated by analyzing other peanut allergens (Ara h 2 (250 ng·mL^−1^) and Ara h 6 (5.0 ng·mL^−1^)) and the egg allergen Gal d 2 (180 mg·mL^−1^). These non-target allergens were selected based on typical ingredients indicated on product labels. The results displayed in Figure 3D indicated that the presence of other non-specific proteins even in the absence of Ara h 1 did not significantly affect the analytical signal. The electroanalytical behavior also supports the specificity of the matched antibody pair chosen for the immunosensors development towards Ara h 1.

### 3.5. Quantification of Ara h 1 in Raw Peanuts and Analysis of Commercial Pre-Packed Food Samples

**Figure 4:**
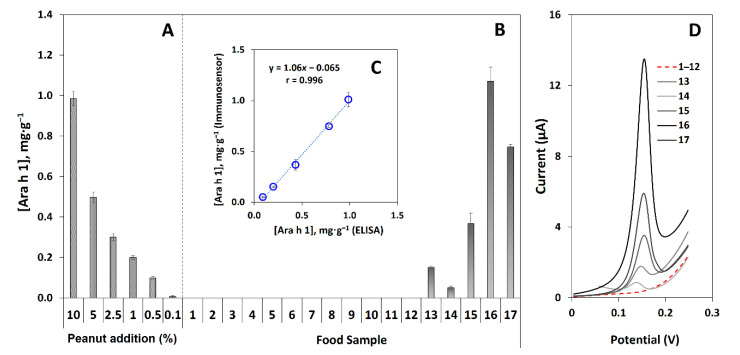
(**A**) Addition of decreasing amounts of peanut (10, 5.0, 2.5, 1.0, 0.50, and 0.10% (*m*/*m*)) in a food sample (biscuit without peanuts). (**B**) Quantification (mg·g^−1^) of Ara h 1 in food samples: 1—wheat flour, 2—oat, 3—lupine, 4—pea, 5—soybean, 6—almond, 7—hazelnut, 8—energy bar (no peanut), 9—biscuit (no peanut), 10—water cracker, 11—oatmeal cookie, 12—whole-grain cereal, 13—granola, 14—muesli, 15—protein bar (5% peanut), 16—protein bar (12% peanut), 17—peanut and pineapple cookie (8% peanut). (**C**) Correlation plot between the results of the developed sensor and ELISA. (**D**) Representative LSV voltammograms of sample analysis. Error bars are the standard deviations of three replicates.

Allergen-free biscuits were selected to perform recovery studies (applied due to the absence of certified reference material). These biscuits were selected as a model food since their composition includes several common ingredients and other allergens (wheat flour (gluten), oat flakes, vegetable fat, powdered full cream milk). Powdered biscuits (1 g) were analyzed after spiking with increasing concentrations of Ara h 1 (0, 100, 250, and 500 ng·mL^−1^). As expected, the non-spiked sample showed no significant differences compared to the blanks. The recoveries for the other concentrations were 90.2%, 96.4%, and 93.9%, respectively, indicating that accurate results were achieved. Moreover, considering the current legislation requirements to mention or advertise the presence of allergen traces, biscuit samples (1 g) were mixed with increasing amounts of raw peanuts (0, 0.10, 0.50, 1.0, 2.5, 5.0, and 10% (*m*/*m*)). The obtained data (Figure 4A) demonstrate that at least 0.10% of peanut (1 mg peanut per 1 g sample) was measurable, thus allowing the trace analysis of peanuts. Ara h 1 was also quantified in a commercial raw peanut sample. The obtained result (4.29 ± 0.16 mg·g^−1^) is lower compared to our previously reported electrochemical immunosensors [18,19].

To evaluate the applicability of the developed sensor, pre-packed food samples and ingredients were analyzed. These were obtained from local supermarkets and their selection was based on foodstuffs to be consumed in breaks between meals (e.g., as a snack). They include vegetables and other food allergens (wheat flour, lupine, pea, soybean, almond, hazelnut), products that do not contain peanuts (biscuit, energy bar), products that may contain peanut traces (water cracker, oatmeal cookie, whole-grain cereal, granola, muesli), and products containing known amounts of peanuts (protein bars and peanut and pineapple cookie).

The quantified amount in the samples is presented in Table 1 and displayed in Figure 4A,B. Firstly, for the ingredients under study (samples 1, 2, 3, 4, 5, 6, 7) no significant differences between the *i*_p_ values and the blank signal were noticeable, thus indicating that their presence does not interfere with the analysis. Also, in the wide range of samples containing the warning “May contain traces of peanuts” (samples 10, 11, 12, 13, 14), only granola and muesli (13 and 14) gave positive results and confirmed the presence of peanut below 1.0% and 0.50%, respectively, thus confirming the presence of peanut traces.

In addition, the Ara h 1 amount detected in products that contain peanuts (samples 15, 16, 17) is in agreement with both the results obtained in the recovery study and the value reported on the product label. The obtained data confirm that the present approach can be an effective tool to analyze low amounts of the target allergen since even in these cases small signal variations were measurable. Quantification of Ara h 1 (mg·g^−1^) was performed using the developed immunosensor and validated with an ELISA method. The correlation values are shown in Figure 4C and representative voltammograms are presented in Figure 4D. The average amount, standard deviation, relative deviation (%), and the applied dilution factor are presented in Table 2. Comparing the results of both assays, a relative deviation of less than 15% was obtained, thus confirming the accuracy of the results of the developed immunosensor.

To date, the analysis of food allergens using a nanodiamond-coated SPCE has not yet been described. The present work reports an SPCE/ND platform with an increased electroactive area, allowing the screening of Ara h 1 in peanut-containing foodstuff and products thereof, with highly accurate results, able to detect at least 0.10% (m/m) of peanut. Compared to previously published electrochemical immunosensors for Ara h 1 quantification (Table 3), several nanomaterials such as SWCNT [16], gold nanoparticles [18], and Quantum Dots [19] were employed to improve the sensor’s performance. Nonetheless, their main drawback is the lack of proficiency to validate the presence or absence of traces in commercially available products and/or distinguishing between the matrix effect and the presence of low amounts. Additionally, the 2-week storage stability allows for timely preparation of the platforms and thus the reduction of reagents and handling time. Our electrochemical SPCE/ND immunosensor overcomes these limitations and the accurate analysis can be helpful to avoid eventual acute reactions in hypersensitive individuals.

The application of a 3D nano-sized material (nanodiamonds) on the transducer surface favored the efficient antibody immobilization on the nanomaterials´ surface due to its inherent characteristics. Also, for the effective allergen detection, applying NDs evidence that a reduced effect (at a negligence level) of the sample matrix is noticeable. Indeed, the suitability to track foodstuffs that contain traces of Ara h 1 alongside the environmentally friendly nanomaterial dispersion strategy contemplates major issues addressed by the novelty of the system.

## 4. Conclusions

Monitoring *Arachis hypogaea* in pre-packaged food is critical to assure food safety and avoid risks to peanut-allergic patients and hypersensitive individuals. Hence, a highly sensitive biomodified nanodiamond-coated screen-printed carbon electrode was applied as the transducer in an electrochemical immunoassay. The transducer’s surface was characterized by voltammetric analysis and an enhancement in current intensity was noticeable due to the increase in the electroactive surface area. Scanning Electron Microscopy showed that the nanodiamonds (11.2 ± 0.9 nm) have a broad distribution on the WE’s surface with a round shape.

A sandwich-type immunoassay was developed with a total assay time of 2 h 20 min (with a hands-on time of about 10 min), a linear range between 25 and 500 ng·mL^−1^, and a very low LOD (0.78 ng·mL^−1^). Results of Ara h 1-spiked biscuits showed good recoveries, reproducibility, and precision. The sensor was successfully applied to the analysis of pre-packed food samples and ingredients, providing accurate results and allowing the quantification of the target allergen, even in trace amounts.

## Figures and Tables

**Table 1:** Experimental variable optimization for the voltammetric immunosensor.

Experimental Variable	Selected Parameter/Value	
**Nanomaterial** (SWCNT, MWCNT, rGO, ND)	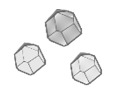	ND
**[ND] (mg·mL^−1^)** (1.0, 0.50, 0.25, 0.10, 0.05, 0.025)		0.10
**[CAb] (µg·mL^−1^)** (5.0, 10, 25)	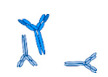	10
**DAb (Dilution)** (1000×, 500×, 250×)	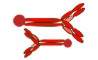	250×
**S-AP (Dilution)** (100,000×, 150,000, 200,000×, 250,000×)	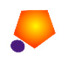	200,000×
**Assay Format** (Step-by-step, Ara h 1 + DAb, DAb + S-AP)	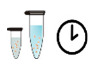	Step-by-step (Format 1)
**Assay time**	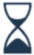	2 h 20min

**Table 2:** Results using the voltammetric SPCE/ND immunosensor and a commercial ELISA kit for quantification of Ara h 1 (mg·g^−1^), relative standard deviations, relative deviation (%), and sample dilution factor.

Food/Ingredient	Ara h 1 (mg·g^−1^)		Relative Deviation (%)	Sample Dilution
	Immunosensor	ELISA		
**Wheat flour**	ND	ND	ND	100×
**Oat**	ND	ND	ND	
**Lupine**	ND	ND	ND	
**Pea**	ND	ND	ND	
**Soybean**	ND	ND	ND	
**Almond**	ND	ND	ND	
**Hazelnut**	ND	ND	ND	
**Energy Bar** (No peanut)	ND	ND	ND	
**Biscuit** (No peanut)	ND	ND	ND	
**Water Cracker** (May contain peanut)	ND	ND	ND	
**Oatmeal cookie** (May contain peanut)	ND	ND	ND	
**Whole-grain cereal** (May contain peanut)	ND	ND	ND	
**Granola** (May contain peanut)	0.18 ± 0.01	0.20 ± 0.01	−11.1	
**Muesli** (May contain peanut)	0.08 ± 0.02	0.09 ± 0.01	−8.60	
**Protein Bar** (5% peanut)	0.37 ± 0.05	0.40 ± 0.04	−7.50	1000×
**Protein Bar** (12% peanut)	1.07 ± 0.07	0.95 ± 0.05	12.6	
**Peanut and Pineapple Cookie** (8% peanut)	0.75 ± 0.01	0.78 ± 0.03	−2.60	
**Raw peanut**	4.29 ± 0.16	4.33 ± 0.31	−0.92	
**Peanut Butter**	4.85 ± 0.26	4.70 ± 0.41	3.19	

ND—Not Detected.

**Table 3:** Comparison of electrochemical immunosensors for Ara h 1 analysis.

Biosensor Construction and Detection Technique	Nanomaterial	LOD	Sample	Ref
SPCE/NDs with CAb immobilized by physical adsorption. Sandwich-type assay, AP used as label. Detection through LSV.	NDs	0.78 ng·mL^−1^	Biscuits, crackers, cookies, cereals, energetic/protein bars	This work
AuE/11-MUA with CAb immobilized through covalent binding (EDC/NHS). Label-free assay, [Fe(CN)_6_ ]^3−/4−^ used for detection. EIS employed as electrochemical technique.	—	0.3 nM	n.d.	[15]
Silicon wafer/SWCNT with CAb covalently immobilized (1-PBSE). Label-free assay. Analysis performed by LSV.	SWCNT	1.0 ng·mL^−1^	n.d.	[16]
SPCE/MBs with CAb immobilized through covalent binding (EDC/NHS). Sandwich-type assay, HRP used as label. Amperometry was elected for detection.	MBs	6.3 ng·mL^−1^	Food extracts, saliva	[17]
SPCE/AuNP with CAb immobilized by chemisorption. Sandwich-type assay, AP used as label. Detection through LSV.	AuNP	3.8 ng·mL^−1^	Cookies, chocolate	[18]
Bare SPCE with CAb immobilized through physical adsorption. Sandwich-type assay, QDs used as label. DPV used for detection.	QDs	3.5 ng·mL^−1^	Cookies, cereal, protein bars	[19]

1-PBSE—1-pyrenebutanoic acid succinimidyl ester; 11-MUA—11-mercaptoundecanoic acid; AuE—gold electrode; AP—alkaline phosphatase; CAb—capture antibody; DPV—differential pulse voltammetry; EIS—electrochemical impedance spectroscopy; EDC—N-(3-dimethylaminopropyl)-N′-ethylcarbodiimide hydrochloride; HRP—horseradish peroxidase; LSV—linear sweep voltammetry; MBs—Magnetic Beads; NHS—N-hydroxysulfosuccinimide; NDs—nanodiamonds; QDs—Quantum Dots; SPCE—screen-printed carbon electrode; SWCNT—single-walled carbon nanotubes; n.d.—no data.

## Data Availability

Not applicable.

## Supplementary Materials

The following supporting information can be downloaded at: https://www.mdpi.com/article/10.3390/bios12060429/s1, Figure S1: Correlation plots between *i*_pa_ and υ^1/2^ for bare SPCE (blue line) and SPCE/ND (red line). Cyclic voltammograms recorded for (B) bare SPCE and (C) SPCE/ND. Scan rates vary from 0.05 to 1.0 V s^−1^; Figure S2. (A) Evaluation of non-specific adsorptions (control assay: absence and presence of Ara h 1) and study of non-specific interactions in the absence of the assay´s immunoreagents (CAb, DAb, S-AP) and the metalloenzymatic label (3-IP, AgNO_3_). (B) Representative CV voltammograms and (C) LSV voltammograms. Experimental conditions: Ara h 1 (0 and 250 ng·mL^−1^), 3-IP/Ag^+^ (1.0 × 10^−3^ M/4.0 × 10^−4^ M); Optimization of the immunoassay construction; Figure S3. Results of the optimizations: (A) concentration of capture antibody (CAb: 5.0, 10, and 25 µg·mL^−1^); (B) detection antibody dilution factor (DAb: 1000×, 500×, and 250×); (C) enzyme conjugate dilution factor (S-AP: 100,000×, 150,000×, 200,000×, and 250,000×); (D) Format assays (Format 1: step-by-step assay, Format 2: previously mixture step of target analyte (Ara h 1) and DAb; Format 3: previously mixture step of DAb and S-AP, Format 4: previously mixture step of Ara h 1, DAb and S-AP in a single assay step; (E) Format 1 assay time (A: Ara h 1, 60 min; DAb, 60 min; S-AP, 30 min; (B) Ara h 1, 60 min; DAb, 30 min; S-AP, 30 min; (C) Ara h 1, 30 min; DAb, 60 min; S-AP, 30 min). Other conditions: Ara h 1 (0 and 250 ng·mL^−1^), 3-IP/Ag^+^ (1.0 × 10^−3^ M/4.0 × 10^−4^ M).
