# Supplementary material for: Tracking Arachis hypogaea Allergen in Pre-Packaged Foodstuff: A Nanodiamond-Based Electrochemical Biosensing Approach
Source: Biosensors (Basel). 2022 Jun 18;12(6):429. doi: 10.3390/bios12060429 (PMC9221418; doi:10.3390/bios12060429)
Supplement: Supplementary file 1 [file biosensors-12-00429-s001.zip › biosensors-1754856-supplementary.pdf]

biosensors

MDPI

Supporting Material

Tracking *Arachis hypogaea* allergen in pre-packaged foodstuff: a nanodiamond-based electrochemical biosensing approach

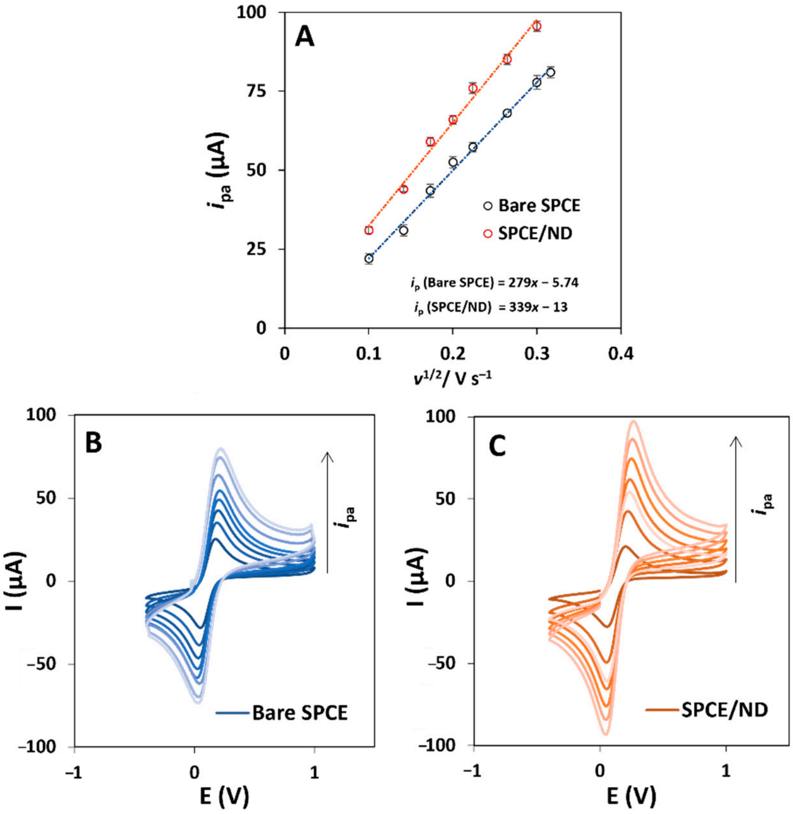

Figure S1. (A) Correlation plots between i_{pa} and $\upsilon^{1/2}$ for bare SPCE (blue line) and SPCE/ND (red line). Error bars are the standard deviation of three replicates. Cyclic voltammograms recorded for (B) bare SPCE and (C) SPCE/ND. Scan rates vary from 0.05 to 1.0 V s^{-1}.

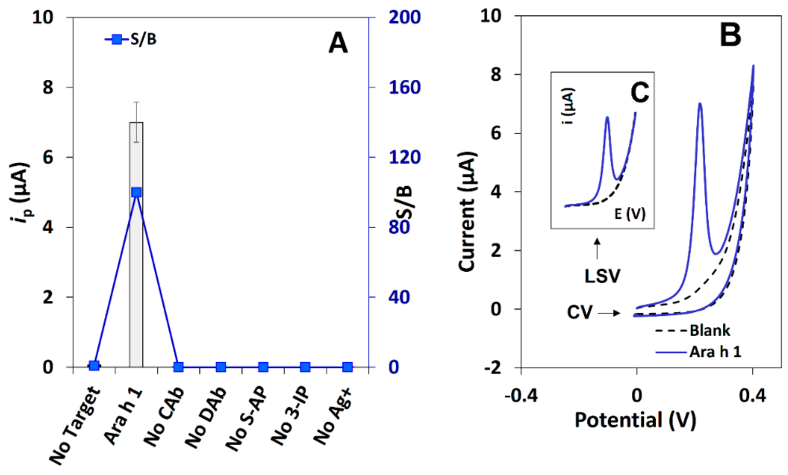

Figure S2. (A) Evaluation of non-specific adsorptions (control assay: absence and presence of Ara h 1) and study of non-specific interactions in the absence of the assay's immunoreagents (CAb, DAb, S-AP) and the metalloenzymatic label (3-IP, $AgNO_3$). **(B)** Representative CV voltammograms and **(C)** LSV voltammograms. Experimental conditions: Ara h 1 (0 and 250 ng·mL^{-1}), 3-IP/Ag^+ (1.0×10^{-3} M / 4.0×10^{-4} M). Error bars are the standard deviations of three replicates.

Optimization of the immunoassay construction

To develop an efficient immunosensing strategy, prior optimizations were performed. The typically employed blocking step (usually with BSA or Casein as blocker reagents) was not introduced in the present immunoassay construction since the sequential immunoassay steps were carried out using solutions previously prepared in a T1-BSA 1.0% (m/V), thus allowing the blockage of non-specific interactions along with incubation steps.

The first phase of the immunoassay development consisted of the optimization of the antibody concentrations using the following assay conditions: for a fixed DAb dilution (500×), distinct CAb concentrations were tested (5.0, 10, and 25 $\mu g{\cdot}mL^{-1}$). The optimum parameter/value was selected according to the signal-to-blank (S/B) ratio for Ara h 1 (0 $ng{\cdot}mL^{-1}$ and 250 $ng{\cdot}mL^{-1}$) (Figure S3A). CAb 10 $\mu g{\cdot}mL^{-1}$ was chosen to proceed with the study and distinct DAb dilutions were tested (1000×, 500×, and 250×) (Figure S3B). A DAb dilution of 250× was found to be the optimum value. Using the optimum conditions (CAb 10 $\mu g{\cdot}mL^{-1}$ and DAb dilution of 250×), the enzyme conjugate (S-AP) dilution was tested (100,000×, 150,000, 200,000×, and 250,000×). The optimum result was obtained for S-AP 200,000× dilution as shown in Figure S3C.

In a second phase, the immunoassay format was tested as follows: Format 1—step-by-step (Ara h 1, 60 min; DAb, 60 min; S-AP, 30 min); Format 2—pre-incubation of Ara h 1 + DAb (Ara h 1 + DAb, 60 min; S-AP, 30 min); Format 3—pre-incubation of DAb + S-AP (Ara h 1, 60 min; DAb + S-AP, 60 min); Format 4—pre-incubation of Ara h 1 + DAb + S-AP (Ara h 1 + DAb + S-AP, 60 min). After each assay format, the enzymatic reaction of 3-IP/Ag^+ ($1.0{\times}10^{-3}$ M / $4.0{\times}10^{-4}$ M) occurred for 20 min. The obtained i_P values (Figure S3D) show a reduced blank signal, thus demonstrating no cross-reactivity among the assay biomolecules and good performance for Format 1 (step-by-step). Furthermore, to reduce the assay time of Format 1, the following incubation times were tested: (A) Ara h 1, 60 min; DAb, 60 min; S-AP, 30 min; (B) Ara h 1, 60 min; DAb, 30 min; S-AP, 30 min; (C) Ara h 1, 30 min; DAb, 60 min; S-AP, 30 min. The results, displayed in Figure S3E, show similar S/B values for tests (A) and (C); however, a higher i_P value was observed for test (C) with a reduction of 30 min in the incubation time of Ara h 1. Thus, using Format 1 (step-by-step) and the incubation times of test (C), the total assay time was 2h20 min.

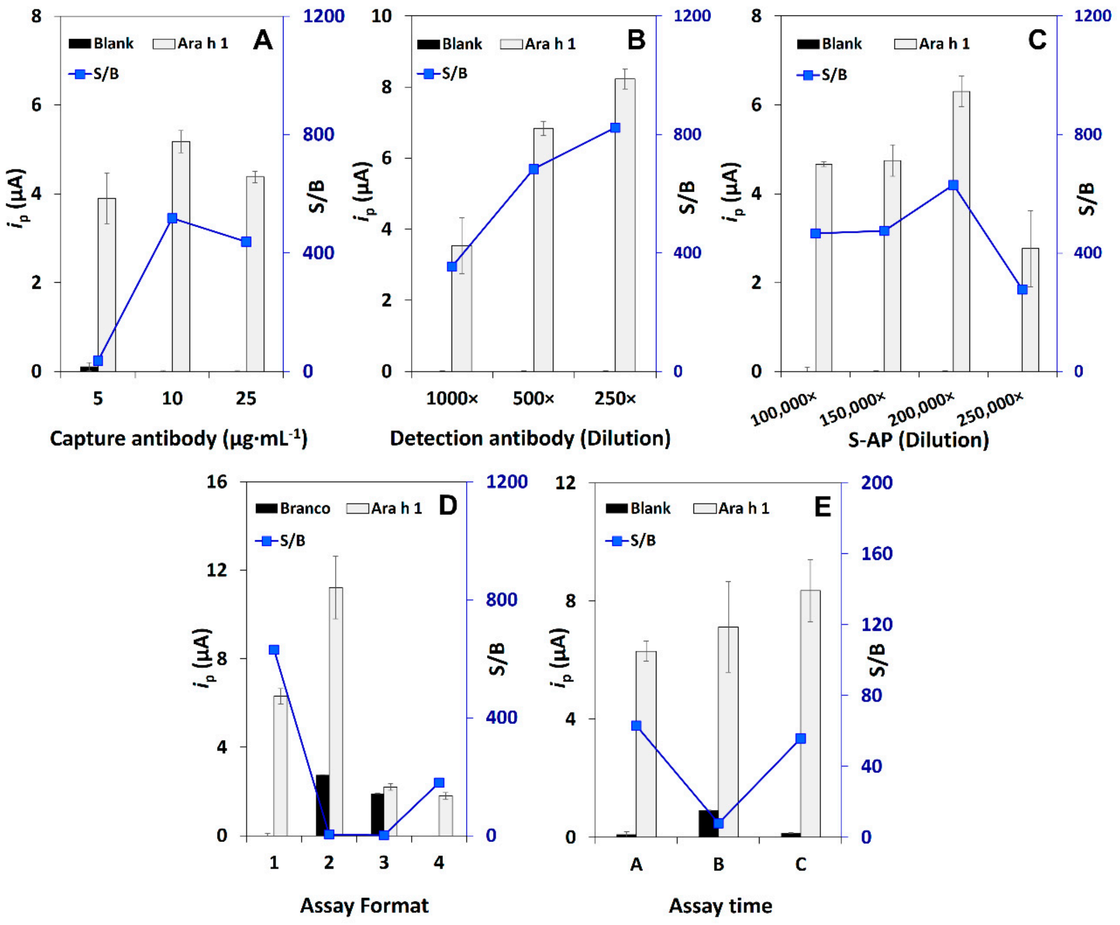

Figure S3. Results of the optimizations: **(A)** concentration of capture antibody (CAb: 5.0, 10, and 25 μg·mL^{-1}); **(B)** detection antibody dilution factor (DAb: 1000×, 500×, and 250×); **(C)** enzyme conjugate dilution factor (S-AP: 100,000×, 150,000×, 200,000×, and 250,000×); **(D)** Format assays (Format 1: step-by-step assay; Format 2: previously mixture step of target analyte (Ara h 1) and DAb; Format 3: previously mixture step of DAb and S-AP; Format 4: previously mixture step of Ara h 1, DAb, and S-AP in a single assay step; **(E)** Format 1 assay time (A: Ara h 1, 60 min; DAb, 60 min; S-AP, 30 min; (B) Ara h 1, 60 min; DAb, 30 min; S-AP, 30 min; (C) Ara h 1, 30 min; DAb, 60 min; S-AP, 30 min). Other conditions: Ara h 1 (0 and 250 ng·mL^{-1}), 3-IP/Ag^+ (1.0×10^{-3} M / 4.0×10^{-4} M). Error bars as the standard deviation of three replicates.
